# Carboxyl-Assisted Synthesis of Nitrogen-Doped Graphene Sheets for Supercapacitor Applications

**DOI:** 10.1186/s11671-015-1031-z

**Published:** 2015-08-20

**Authors:** Bingqiao Xie, Ying Chen, Mengying Yu, Xiang Shen, Hanwu Lei, Ting Xie, Yong Zhang, Yucheng Wu

**Affiliations:** Engineering Research Center of Nano-Geomaterials of Ministry of Education, China University of Geosciences, Wuhan 388 Lumo RD, Wuhan, 430074 China; Bioproducts Science and Engineering Laboratories, Washington State University, 2710 Crimson Way, Richland, WA 99352 USA; Key Laboratory of Advanced Functional Materials and Devices of Anhui Province, Hefei, 230009 China; School of Materials Science and Engineering, Hefei University of Technology, Hefei, 230009 China

**Keywords:** N-doped graphene, Carboxylation, Electrochemical, Supercapacitor

## Abstract

The high ratio of pyridinic and pyridone N-doped graphene sheets have been synthesized by functionalizing graphene oxide (GO) with different oxygen groups on its surface. The typical N-doped graphene was determined to be ~3–5 layers by transmission electron microscopy (TEM) and atomic force microscopy (AFM), and the nitrogen content was measured as 6.8–8 at. % by X-ray photoelectron spectroscopy (XPS). The structure of the N-doped graphene with different surface functional groups was characterized by Raman spectroscopy. The research result indicates that the carboxylation of GO is the key factor to obtain pyridinic and pyridone N types during the N atom doping process. Compared to general N-doped graphene, the electrochemical test shows that specific capacitance of the GO-OOH-N sample reaches up to 217 F/g at a discharge current density 1 A/g and stable cycling performance (keep 88.8 % specific capacitance after 500 cycles at the same discharge current density) when applied to the supercapacitor electrode materials.

**Electronic supplementary material:**

The online version of this article (doi:10.1186/s11671-015-1031-z) contains supplementary material, which is available to authorized users.

## Background

Graphene is an allotrope of a new-type carbon with two-dimensional carbon atoms tightly packed into honeycomb lattice [1]. The special intrinsic structure exhibits excellent mechanical, electrical, and chemical properties [2–4], which have shown promising wide applications in various fields [5–10]. However, chemically inert and hydrophobic feature of graphene surface makes it weakly interactive with other media and poorly soluble in water and common organic solvents. In addition, graphene is a semimetal with zero bandgap which is the major obstacle limiting its utilization for many device applications that need variable electronic bandgap [11].

By doping nitrogen into graphene, the bandgap can be opened, which makes it possible to adjust the conductivity type and the electronic structure of graphene; thus, the physical and surface performance of graphene can be modulated [1, 11, 12]. Additionally, by introducing nitrogen atoms into the carbon grid of graphene, the active sites for adsorbing metal ions and redox reactions can be increased, which could enhance the interaction between metal particles and graphene, also the ability to trigger Faraday reaction [13]. However, the N content of graphene cannot be infinitely improved. On the one hand, high doping content of N can lead to the formation of carbon nitride films which has extremely low electrical conductivity; on the other hand, the increasing defects caused by nitrogen doping inevitably lead to the decline of the intrinsic features of graphene [14, 15].

In general, the graphene and nitrogen atoms could be combined together in four ways [16–21]. Considering the *sp*^2^-hybridized carbons will hinder the N atoms doping into graphene lattice, the quaternary-type N is difficult to form, and often with lower N content or even the failure of doping [22]. Oxidized pyridinic nitrogen is also hard to achieve a high doping content (few further discussions on its acting mechanism were referred in the paper). Besides, the pyridinic nitrogen and pyridone nitrogen (or pyrrolic nitrogen) are easily formed on the graphene surface and also proven to be valuable electrochemical active sites. Thus, the effective regulation of pyridinic, pyrrolic, or pyridone N in graphene-like network together with a well-defined pore structure is very important for accelerating application of carbon-based materials in energy storage fields such as lithium-ion batteries [23], fuel cells [24, 25], and supercapacitors [26].

Supercapacitors are new energy storage device, and they have many advantages attracting tremendous attentions, such as high power density, excellent cycling stability, and fast charge/discharge capability [27]. Graphene is commonly used as electrodes in electrochemical double-layer capacitors (EDLC) because of its high surface area and conductivity. In order to improve the electrochemical properties of EDLC, decorating the graphene with N is a feasible way [28].

The effect of N doping types of graphene on supercapacitor performances has been previously investigated [5, 26, 29, 30]. The research found that pyridone nitrogen atoms can be considered as pyridine nitrogen atoms with one adjacent carbon atom that adsorbs a hydroxy group, and the conversion between pyridone and pyridine nitrogen atoms through Faraday processes is reasonable [28, 31]. Through the redox reaction between them, the pseudocapacitance is increased, which contributes to the increase of the capacity of the supercapacitors. Recently, pyridinic-like holes in situ formed during the reduction and nitrogen doping processes of GO have been reported in many papers [18, 31, 32]. These micropore/mesopore textures can enhance the charge accommodations and finally endow the electrode material with a high energy density [33]. In addition, quaternary-type nitrogen will help to improve the wettability between the electrolyte and the electrode, thereby enhancing the pseudocapacitance effect. Moreover, quaternary-type nitrogen and oxidized pyridinic nitrogen are able to maintain a stable capacity during fast charging and discharging [28].

In this paper, we obtained the N-doped graphene with different N types and content by adjusting the oxygenic functional groups on the graphene surface. As a result, the graphene with a high percentage of both pyridinic and pyridone N was obtained from carboxylic graphene oxide. The graphene with designed N types exhibits superior capacitance and cycling performance in the electrochemical tests, suggesting that special oxygen functionalization of graphene surface is a promising method to synthesize N-doped graphene with optimized N-doped types for high-performance supercapacitors.

## Methods

### Materials and Equipments

Materials used were as follows: natural flake graphite (graphite, purity 99.8 %, 600 meshes, Qingdao), KMnO_4_ (AR, Sinopharm Chemical Reagent Co., Ltd.), N_2_H_4_**·**H_2_O (80 %, AR, Tianjin Tianli Chemical Reagent Co., Ltd.), H_2_SO_4_ (98 %, AR, Xinyang Chemical Reagent Factory), H_3_PO_4_ (85 %, AR, Sinopharm Chemical Reagent Co., Ltd.), HCl (37 %, Sinopharm Chemical Reagent Co., Ltd.), H_2_O_2_ (30 %, Sinopharm Chemical Reagent Co., Ltd.), C_2_H_5_OH (Sinopharm Chemical Reagent Co., Ltd.), ClCH_2_CO_2_H (Shanghai Taishan Chemical Plant), NH_3_**·**H_2_O (30 %), and home-made secondary deionized water. All medicines were not treated with further purification.

### Characterization Methods

All the graphene and N-doped graphene sheets were characterized with the JOL2010 transmission electron microscope (TEM; accelerating voltage of 200 KV), PHI-5400 X-ray photoelectron spectroscopy (XPS), Rxn1-785 Raman spectrometer (*λ* = 690 nm), and Nanoscope IIIa controller atomic force microscope (AFM).

### Experimental Process

#### Synthesis of Graphene Oxide (GO)

Three grams of natural flake graphite was added into 400 ml of ice-mixed acid (*V*_H2SO4_:*V*_H3PO4_ = 9:1). Then 18 g of KMnO_4_ was slowly added. The temperature was reached to 50 °C and kept at the temperature for 60 h accompanied by magnetic stirring. Finally, the solution was stewed and cooled down to room temperature. After that, deionized water of 300 ml was slowly added for dilution, and 10 ml of H_2_O_2_ and 100 ml of HCl were added to remove impurities. Then a large amount of distilled water was used to make washing and centrifugation, which were repeated for several times until the pH of the solution was neutral. The brown precipitate was frozen and dried for preservation.

#### Synthesis of Carboxylic Graphene (GO-OOH)

The as-prepared GO (0.5 g) was dispersed in 250 ml of distilled water using ultrasonic equipment. Yellow transparent colloidal solution was regained. Then NaOH (10 g) and ClCH_2_CO_2_H (7.5 g) were added, and the solution was stirred for 3 h. After the reaction was complete, the solution was washed repeatedly to remove impurity ions until the neutral state was reached. Vacuum drying was conducted at 60 °C to harvest GO-OOH.

#### Synthesis of N-Doped Graphene Based on GO (GO-N)

Twenty-five milliliters of GO solution (6 mg/ml) was taken, and its pH was adjusted to 10 by NH_3_·H_2_O. Then 20 ml of N_2_H_4_·H_2_O solution was added followed by stirring for 30 min. The solution was then transferred into the reaction vessel and kept for 5 h at 130 °C, and black product was obtained. Centrifugal washing was performed for several times with distilled water and ethanol. N-RGO was finally obtained by vacuum drying at 60 °C.

#### Synthesis of N-Doped Graphene Based on GO (GO-N-180)

The process for preparation is the same as GO-N, except that the react temperature is 180 °C.

#### Synthesis of N-Doped Graphene Based on GO-OOH (GO-OOH-N)

The process for preparation of GO-OOH-N is the same as GO-N, except that the precursor solution GO is replaced by GO-OOH.

### Electrochemical Measurements in a Three-Electrode System

A mixture containing 80 wt % active materials (3 mg), 10 wt % acetylene black, and 10 wt % poly-tetrafluoroethylene (PTFE) was well mixed in *N*,*N*-dimethylformamide(DMF) until they formed a slurry with the proper viscosity, and then the slurry was uniformly laid on a piece of Ni foam about 1 cm^2^ that was used as a current collector and then dried at 80 °C for 2 h. The Ni foam coated with the composite was pressed for 1 min under 1.0 MPa and dried at 120 °C for another 12 h. A Pt electrode and Ag/AgCl electrode filled with saturated KCl aqueous solution were used as the counter electrode and reference electrode, respectively. Cyclic voltammetry (CV), galvanostatic charge/discharge, and electrochemical impedance spectroscopy (EIS) were measured on a CHI760E electrochemical workstation in a three-electrode system. The specific capacitance is calculated according to equation *C* = *It*/*V*, where *I* is the charge/discharge current density (A/g), *t* is the discharge time (*t*), and *V* is the voltage (1 V). The reported specific capacitance and energy density are all normalized to the weight of sample.

## Results and Discussion

**Fig. 1:**
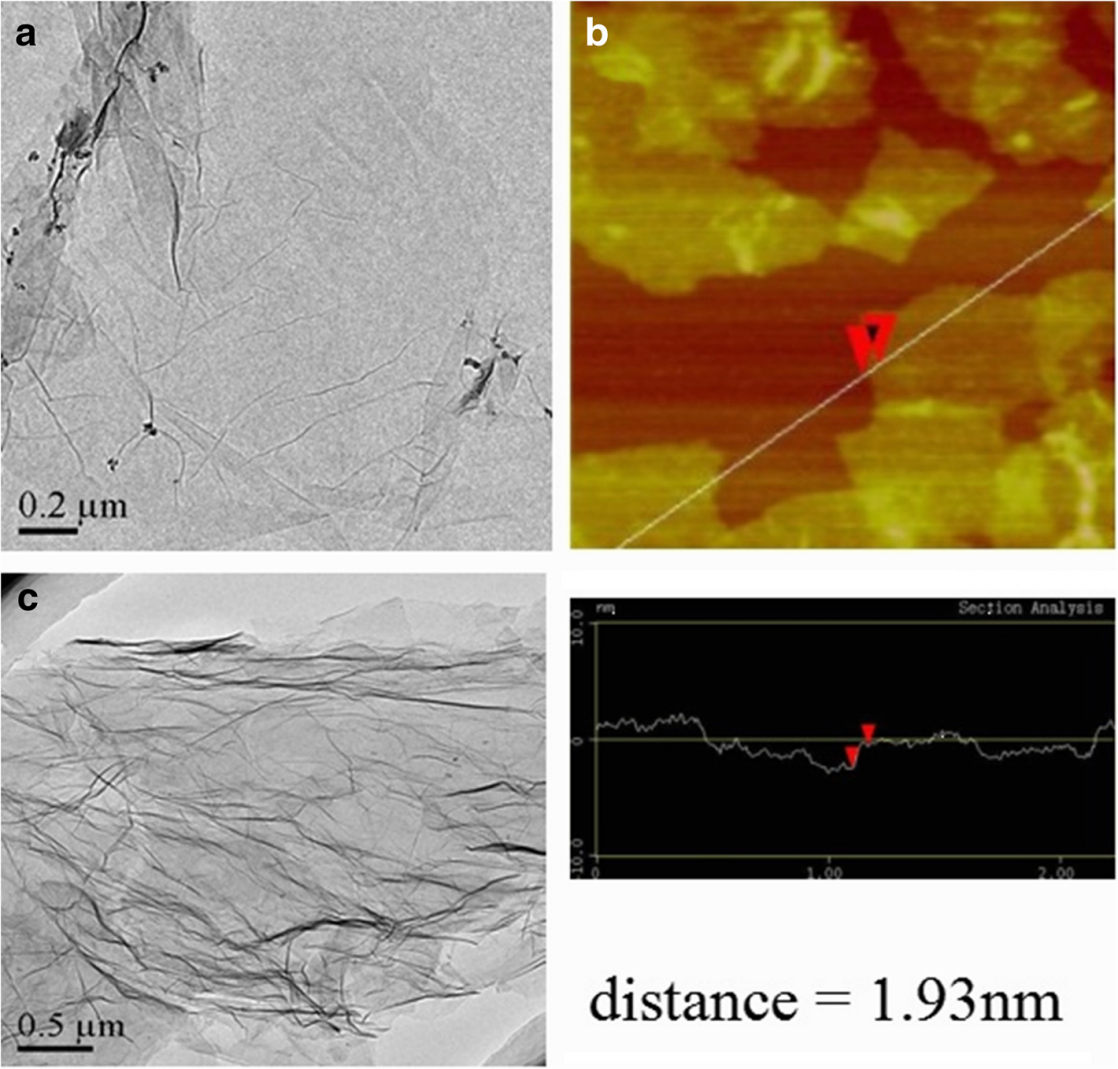
**a, b** is the TEM image and AFM image of graphene oxide; **c** is TEM image of N-doped graphene

Figure 1a shows TEM image of the graphene oxide (GO) with high transparence and displays gauze-like morphology. The GO has slight folds and crimped edges due to the oxygen functional groups that produced defects on the sample surface and edges. The thickness of GO can be determined by AFM (Fig. 1b), which shows that the thickness distribution of GO is relatively uniform with two to three layers [34]. After the nitrogen doping treatment (Fig. 1c), wrinkled degree of the graphene surface aggravated obviously because of the more defect formation and the N atoms induced into the graphene. Notably, the nitrogen-doped graphene with a pre-carboxylation treatment on GO precursor exhibits better disperse ability in ethanol (see Additional file 1: Figure S1), illustrating that adequate oxygen-containing groups were still remained in GO-OOH-N after hydrothermal reduction. The N-doped process and mechanism are similar to the bamboo-shaped feature of nitrogen-doped carbon nanotubes [35].

**Fig. 2:**
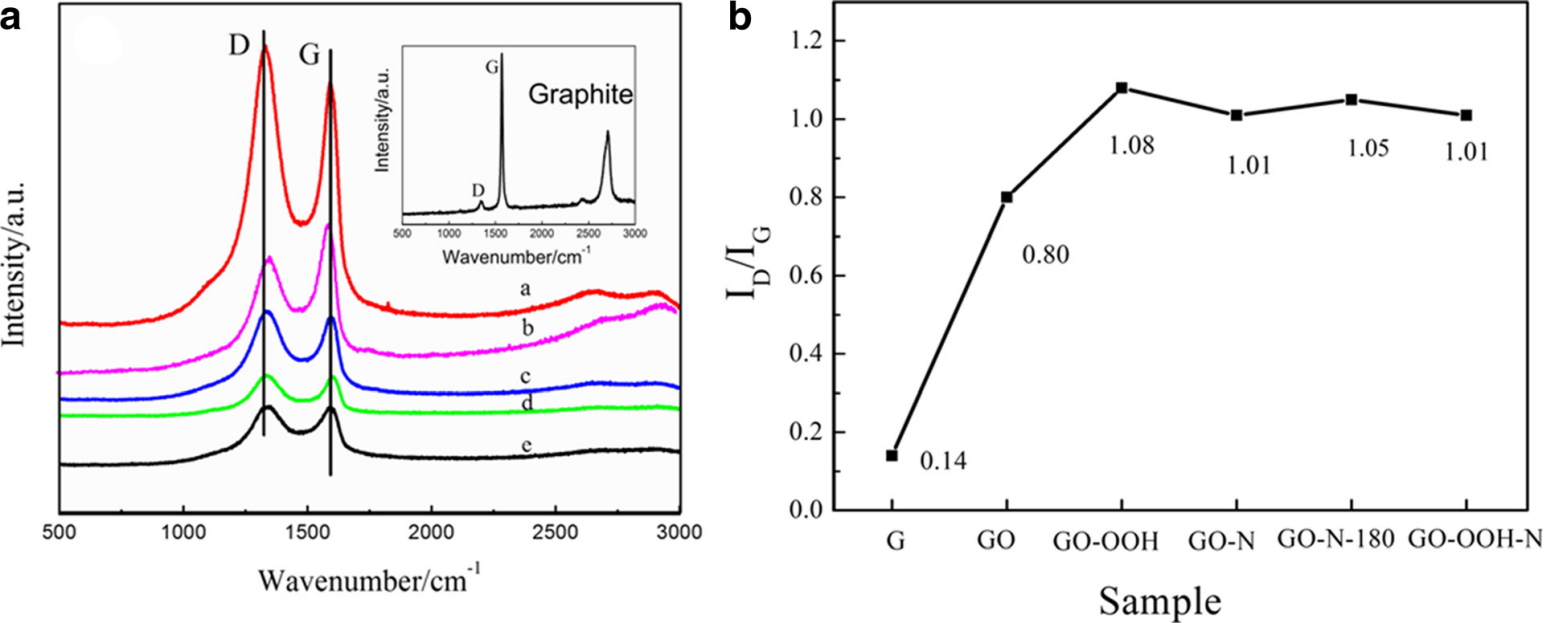
**a** Raman spectra of GO-OOH (*a*), GO (*b*),GO-N-180 (*c*), GO-OOH-N (*d*), GO-N (*e*); **b** *I*_*D*_/*I*_*G*_ value

Figure 2 shows the Raman spectra of natural graphite, GO, GO-OOH, GO-N, GO-N-180, and GO-OOH-N. All spectra show two characteristic peaks of graphitic carbon materials as G band near 1580 cm^−1^ and D band near 1350 cm^−1^, representing the degree of crystallization and the degree of disorder or defect in the samples, respectively. The former is caused by in-plane stretching vibration under the *E*_2*g*_ mode, and the latter is caused by the scattering of phonons at the boundary of the disordered hexagonal Brillouin zone [36]. It can be seen from the inset in Fig. 2a that the crystallization degree of original graphite was very high (*I*_*D*_/*I*_*G*_ = 0.14). After oxidization treatment, the crystallization degree of GO decreased obviously due to the oxygen-containing groups induced. For the carboxylic graphene, broadening of D band indicates more disorder of the structure, which is related to the increase of oxygen content and the size reduction caused by bond breaking in a transition from C-O-C to -COOH. By nitrogen doping and reduction treatment, N-doped graphene samples were obtained, with a small reduction in *I*_*D*_*/I*_*G*_ value (from 1.08 to 1.01). This is the result of the combined action of several factors: (1) reduction of oxygen-containing groups led to decrease of *I*_*D*_*/I*_*G*_ value; and (2) more structural defects of graphene caused by N doping would increase the value of *I*_*D*_*/I*_*G*_; the *I*_*D*_*/I*_*G*_ values of GO-N, GO-N-180, and GO-OOH-N were similar, indicating close defect density of the N-doped samples but different defect type and distribution, as confirmed by XPS results.

**Fig. 3:**
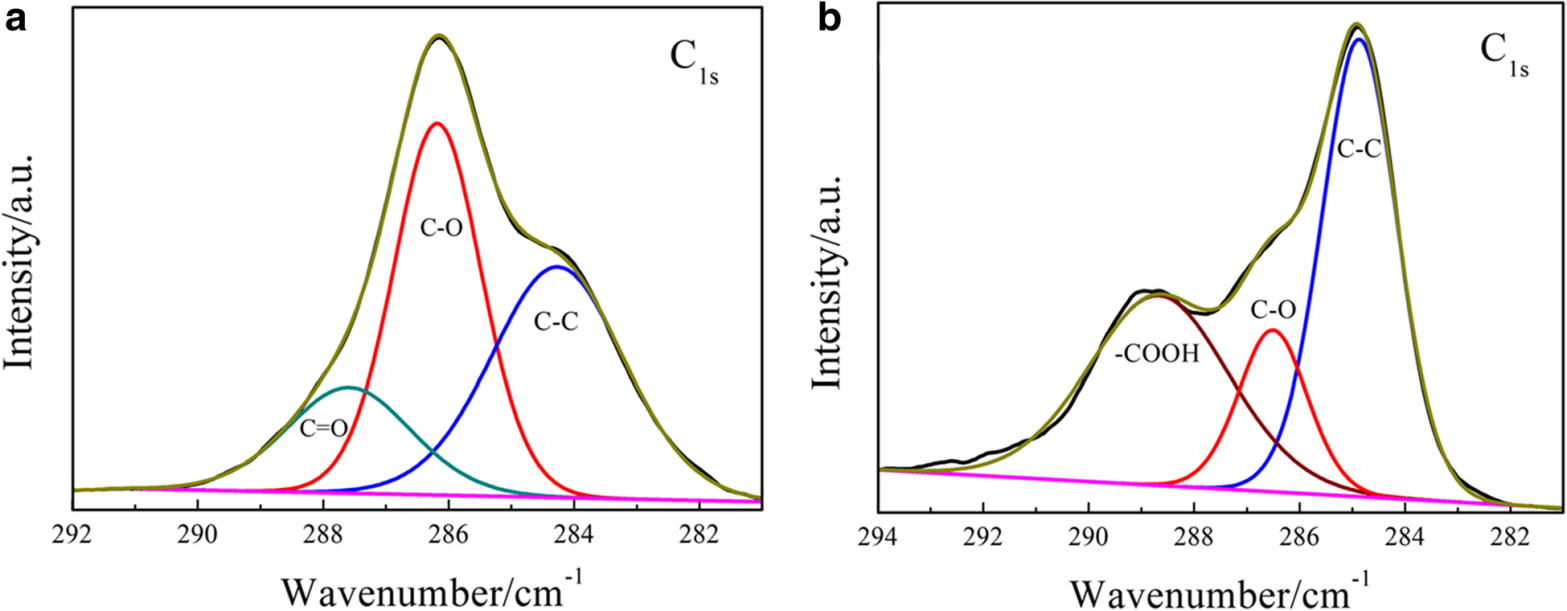
XPS C1s spectra of **a** GO and **b** GO-OOH

As an effective tool to characterize elemental contents and bonding states in the samples, XPS technique can be used to understand the changes in oxygen and nitrogen content and their existing forms in the samples before and after reaction. Figure 3a shows that oxygen-containing groups of the GO sample were mainly composed of epoxy, hydroxyl, and carbonyl groups. After carboxylation of GO, the changes in the content of oxygen-containing groups caused the slight increase of total oxygen content (from 38.6 to 39.7 at. %). It can be seen in Table 1 that the contents of epoxy groups and hydroxyl groups decreased after carboxylation. On the contrary, the content of carboxyl groups increased considerably. Under strong alkaline condition, monochloroacetic acid can transform other oxygen-containing groups into carboxyl groups. Such transformation has been confirmed in previous reports [37, 38]. It should be noted that after the treatment, the intensity of the *sp*^2^ C-C peak was enhanced. The possible reason is that the carboxyl groups with high binding energy were introduced to the edge of graphene, while the epoxyl groups were distributed inside in-plane [31]. The reduction in the content of epoxyl groups was favorable for the recovery of graphitized C-C bond. The carboxylation of GO followed by reduction might be a potential method to obtain high-quality graphene.

**Table 1:** Elemental composition and distribution of type of carbon-containing groups on the surface of GO and GO-OOH

Sample	C (at. % )	O (at. % )	C/O	C Distribution (%)			
				C-C	C-O	C=O	COOH
				(284.3–284.8 eV)	(286.2–286.5 eV)	(287.6 eV)	(288.7 eV)
GO	61.4	38.6	1.6	24.6	26.2	10.6	
GO-OOH	60.3	39.7	1.5	28.7	9.3		22.2

**Fig. 4:**
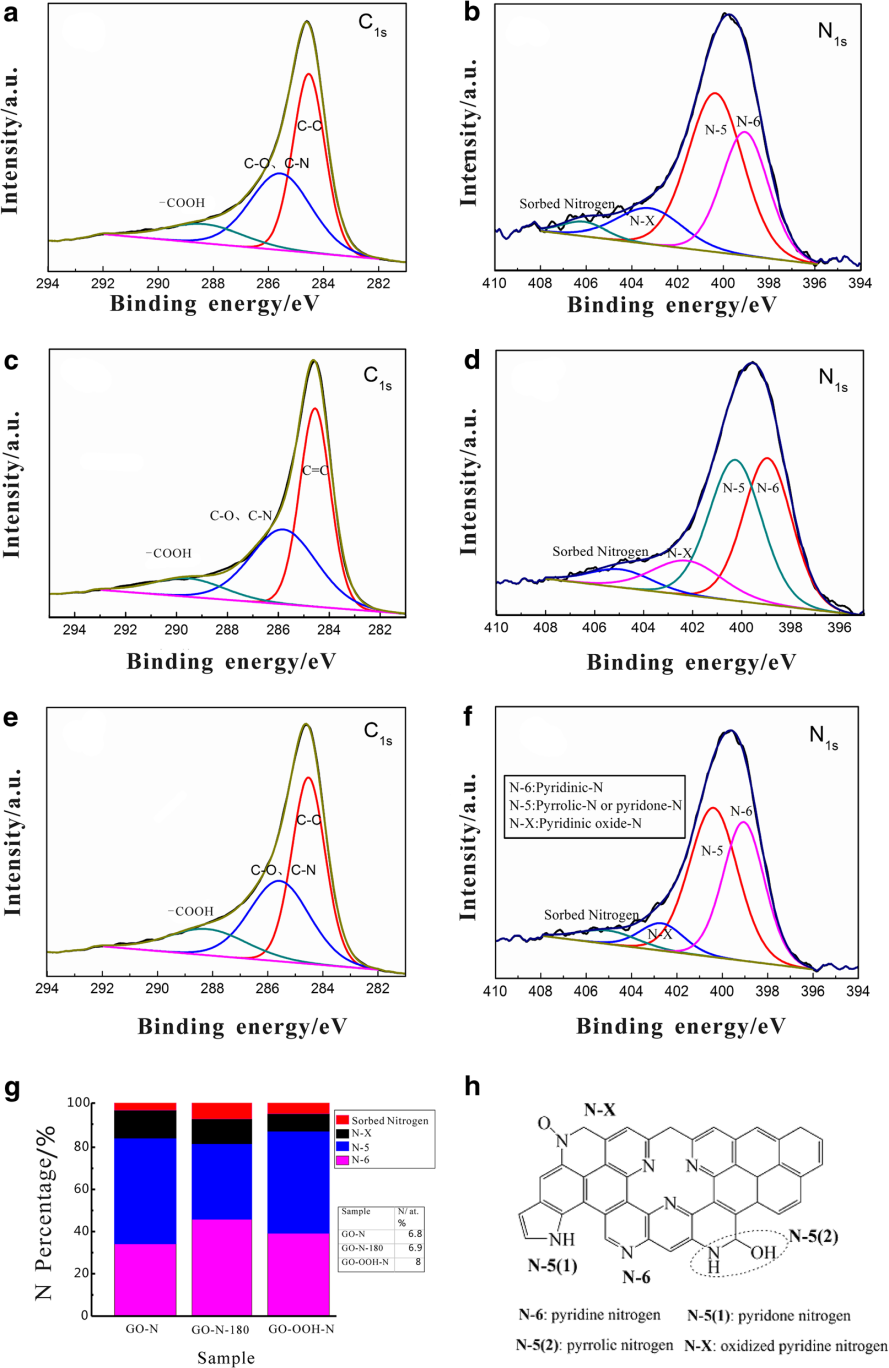
XPS C1s and N1s spectra of GO-N (**a**, **b**), GO-N-180 (**c**, **d**), and GO-OOH-N (**e**, **f**); N-containing groups distribution in N-doped graphene samples (**g**); types of N-containing groups (**h**)

After nitrogen doping, C1s peak of all NG samples shows only one main peak at 284.6 eV and can be divided into three sub-peaks centered at approximately 284.6, 286, and 289 eV (see Fig. 4a, c, e), which belong to C-C, C-O/C-N, and C=O/-COOH, respectively. A relative higher -COOH density of GO-OOH-N in both C1s and O1s (Additional file 1: Figure S3) spectra can partially explain the internal reason of its water affinity feature, which is in agreement with the above experimental phenomena. And these oxygen types are favorable to provide pseudocapacitance for high electrochemical performance [39].

Four N1s sub-peaks could be fitted [31] in Fig. 4: pyridinic nitrogen, pyrrolic or pyridone nitrogen, oxidized pyridinic nitrogen, and adsorbed nitrogen. The peak with the bonding energy at 400–400.6 eV can be attributed to five-membered ring of pyrrole or pyridone structure (pyridone). If the peak is attributed to pyridone, it can be mutually converted with pyridinic nitrogen in specific condition. This is because both oxidized pyridinic nitrogen and pyridone nitrogen are in oxidized states, while pyridinic nitrogen is in reducing state [17].

The details of N-doped graphene under different conditions are shown in Table 2. The total nitrogen content was 6.6 at. % in GO-N ( ignoring the absorbed nitrogen); the content of pyridinic nitrogen (2.3 at. %) was lower than that of pyridone nitrogen (3.4 at. %); there was few oxidized pyridinic nitrogen (0.9 at. %) and no quaternary-type nitrogen appeared. For GO-N-180, the total nitrogen content was 6.4 at. %, and the content of pyridinic nitrogen increased from 2.3 to 3.2 at. %; the content of pyridone nitrogen decreased from 3.4 to 2.4 at. %; oxidized pyridinic nitrogen showed a slight reduction by 0.1 at. %. And the nitrogen content was 7.6 at. % in GO-OOH-N; the content of pyridinic nitrogen increased from 2.3 to 3.1 at. %, and the content of pyridone nitrogen increased from 3.4 to 3.9 at. %; oxidized pyridinic nitrogen decreases by 0.3 at. %. The ratio of pyridinic and pyridone N types is higher than most of the literatures (Additional file 1: Table S3), which reach up to ~88 %.

**Table 2:** Elemental composition and distribution of the type of nitrogen-containing groups on the surface of GO-N and GO-OOH-N samples

Sample	C (at. % )	N (at. % )	N/C (%)	N distribution (at. % )			
				N-6	N-5	N-X	Absorbed Nitrogen
				(398.7–399.1 eV)	(400–400.6 eV)	(402.7–403.2 eV)	(405.0–406.1 eV)
GO-N	83.4	6.8	8.2	2.3	3.4	0.9	0.2
GO-OOH-N	79.1	8.0	10.0	3.1	3.9	0.6	0.4
GO-N-180	84.7	6.9	8.1	3.2	2.4	0.8	0.5

In order to know the relationship between the other oxygen groups and the content and types of N-doped graphene, we also made hydroxy group on graphene surface and the related synthesis method and characterization were in the supporting information. The XPS spectra of G-OH and G-OH-N show that the reduced graphene obtained with 3.9 at. % N doping, in which the content of pyridinic nitrogen, pyridone nitrogen, and oxidized pyridinic nitrogen was 1.2, 2.1, and 0.4 at. %, respectively.(Additional file 1: Figure S2, Additional file 1: Table S2).

**Fig. 5:**
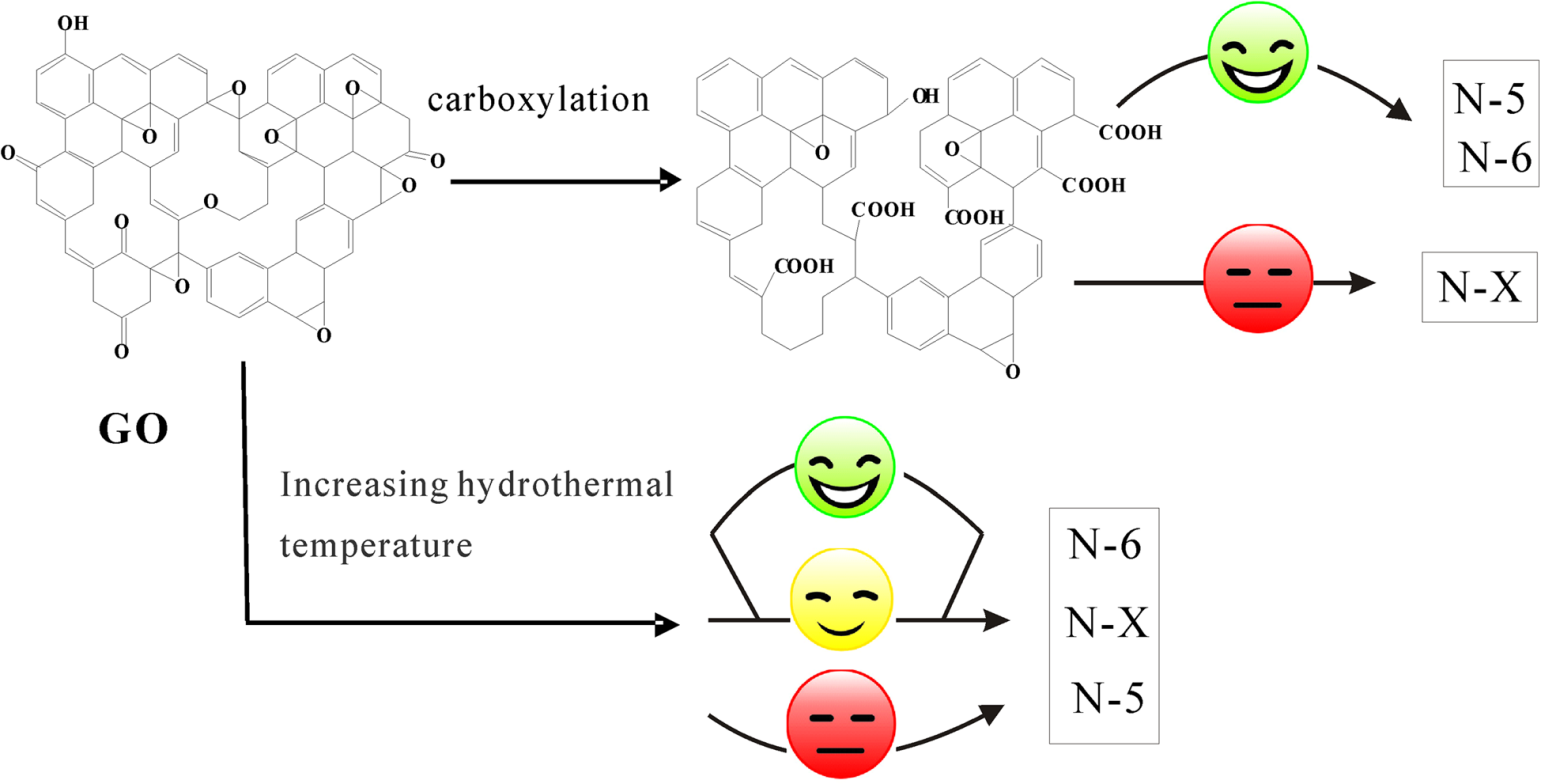
Schematic map of N doping types in GO and GO-OOH

In our experiments, hydrazine hydrate served as both reducing agent and nitrogen source for the treatment of GO, G-OH, and GO-OOH. When GO was used as the precursor material, increasing temperature is favorable for the generation of pyridinic nitrogen but hampers the generation of pyridone nitrogen and oxidized pyridinic nitrogen. This is because both oxidized pyridinic nitrogen and pyridone nitrogen contain oxygen groups. Increasing temperature would favor the reduction reaction which promoted the reduction of oxidized pyridinic and pyridone nitrogen into pyridinic nitrogen [17]. While GO-OOH was employed as the precursor material, the nitrogen content was increased under similar oxidization degree and identical nitrogen doping and reduction treatment conditions. With the reduction in epoxyl groups and the increase in carboxyl groups, the surface activity of graphene was enhanced [40]. Since these carboxyl groups introduced nitrogen into the active sites, the nitrogen content of graphene samples was increased. Moreover, more pyridinic nitrogen and pyridone nitrogen were produced, and the introduction of carboxyl groups had a greater impact on the generation of the former. The influence of experimental factors on nitrogen configuration can be depicted in Fig. 5. Although the high ratio of pyridinic and pyridone nitrogen was also obtained using G-OH as precursor, the total nitrogen content was low. Moreover, the G-OH-N had lower ratio of N/C/ % (4.8 %) than GO-OOH-N (10 %) which indicates that GO-OOH was more effective for N doping in graphene. Thus, the carboxylation of GO is the best choice to obtain high total nitrogen content and designed nitrogen types.

**Fig. 6:**
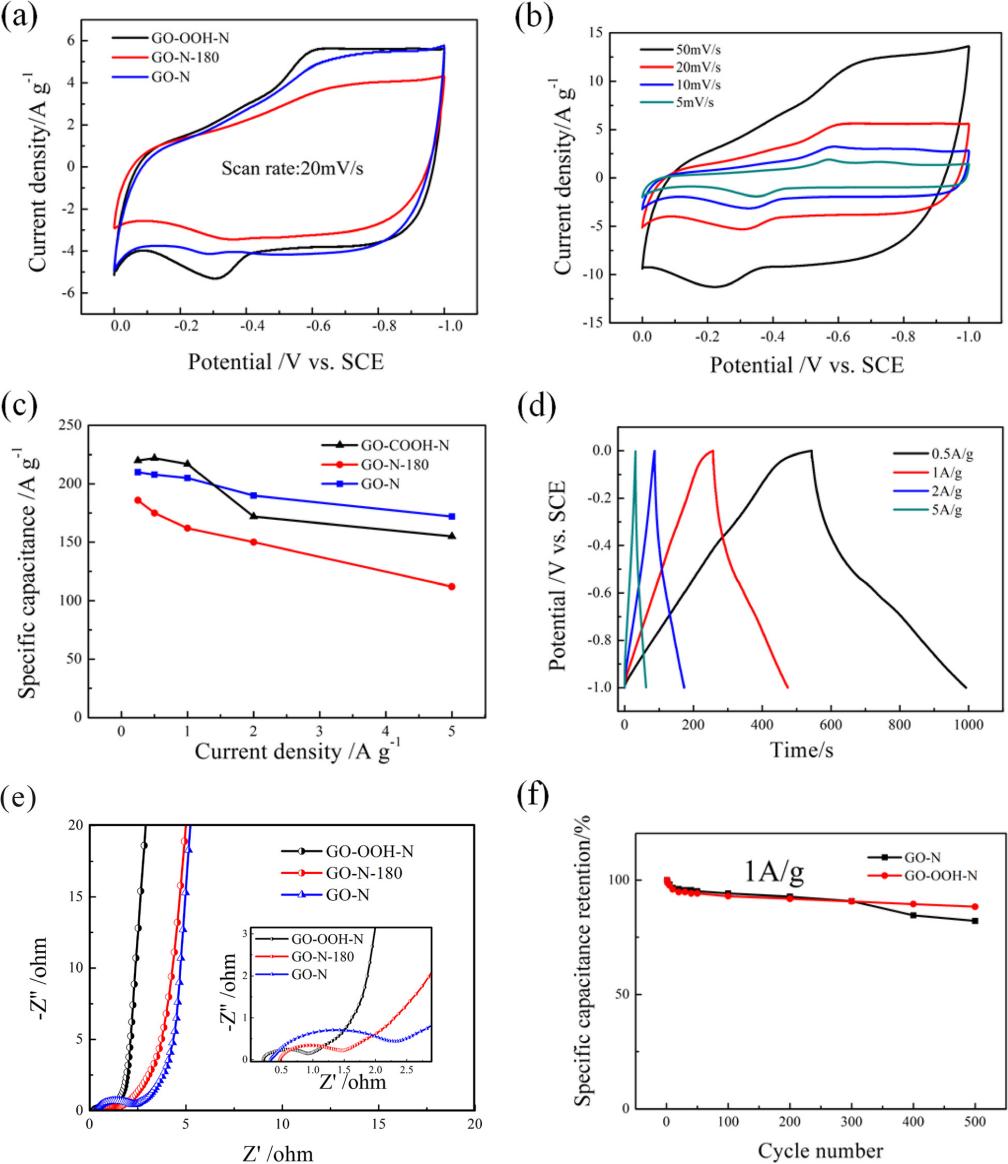
**a** CV curves of samples at 20 mV/s. **b** CV curves of GO-OOH-N at different scan rates. **c** Specific capacitance of samples at different current density. **d** Galvanostatic charge/discharge (GCD) curves of GO-OOH-N. **e** EIS spectrum of samples. **f** Cycling performance of GO-N and GO-OOH-N

The supercapacitor performances of GO-N, GO-N-180, and GO-OOH-N were comparatively investigated in a 6 M KOH solution using a three-electrode configuration. Figure 6a shows the cyclic voltammetry (CV) curves of different samples with the scan rate at 20 mV/s. They all show a good rectangle shape with well-broaden peaks, indicating a capacitive behavior of electrical double-layer capacitance (EDLC) and pseudocapacitance. It is clear that the maximum specific capacitance of GO-OOH-N possesses the largest curves area and the most pronounced pseudocapacitive peaks because of the high content (proportion) of N-5/N-6 and carboxyl groups, which is closely linked with Faradic reaction [26]. Compared to GO-N, the curve area of GO-N-180 electrode decreased apparently due to its higher defect degree. With the scan rate increased, the pseudocapacitive feature of GO-OOH-N remained at CV curves all along from 5 to 50 mV/s (Fig. 6b), indicating a good wettability and easy access of ions to the GO-OOH-N electrode surface. However, the peaks arising from the redox reactions of surface functionalities are slighted reduced at higher scan rates, due to the relatively low charge/discharge kinetics compared to EDLC.

The specific capacitance of the electrode is obtained from the equation: Csp = *It*/△*Em* where Csp, *I*, *t*, △*E*, and *m* are specific capacitance (F/g), constant current (A), discharge time (s), potential window (V), and mass of the active material (g), respectively. As shown in Fig. 6c, the Csp of GO-N, GO-N-180, and GO-OOH-N at 1 A/g is 205, 162, and 217 F/g, respectively, which is in accord with the CV analysis. To probe the potential causes of this increase in capacitance value, we made an investigation on the samples from the point of both chemical composition and physical structure. The negatively charged nitrogen-containing groups (N-6/N-5) can facilitate the electrostatic interaction between K^+^ and N atoms, giving rise to abundant electrolyte ions occupied on the electrode surface [39]. Clearly, the similar curve of GO-N and GO-OOH-N has been observed in BET results (Additional file 1: Figure S4 and Additional file 1: Table S3). The specific surface areas of GO-N and GO-OOH-N are 262 and 223 m^2^/g, respectively. The pore size and distribution are close for both samples (especially for the mesopore), indicating that this markedly enhanced capacitance ability of GO-OOH-N ought to be attributed to the high N content and favored N structures benefiting from an artificial carboxylation on graphene oxide precursor instead of pore structure difference. Unexpectedly, the Csp of GO-OOH-N undergoes a visible drop from 1 to 2 A/g and reaches to a lower capacitance level compared to that of GO-N; this observation may be elucidated by the instability of the pseudocapacitive redox pairs at high current density [41], which can also be proven by the absence of clear mutation in the discharge curve segment of GO-OOH-N at a current density of more than 2 A/g in Fig. 6d. In contrast, GO-N can still exhibit a superior capacitance retention even at high current densities, herein it can be ascribed to the important role of the positively charged groups (N-X) which helps in an electron transfer especially at fast charge/discharge process [18, 28].

The impedance analyses of all products are measured in the frequency range of 100 kHz–0.01 Hz at open circuit potential to evaluate the kinetic and fundamental behavior of electrode materials and are shown in Fig. 6e. The Nyquist plots of N-doped graphene samples are composed of a partial semicircle in high-frequency region and a nearly straight line in low-frequency regions. The semicircle diameter is commonly used for the measurement of the surface properties of the electrode materials and corresponds to the charge transfer resistance. The smaller diameter of GO-OOH-N indicates that it can offer faster charge transportation, which can be contributed to the relative low defect density (indicated in Raman spectrum in Fig. 2b) and the desired N structures as discussed above. The nearly vertical line in the mid- to low-frequency region for all electrode materials reflects that the double-layer structure can be established rapidly, indicating a fast ion transfer kinetics. The excellent dynamic behavior is believed to be closely linked to the good wettability of materials benefiting from the suitable oxygen contents (~10 at. % ) and the hydrophilic groups (i.e., carboxyl groups) incorporated in the carbon networks. In addition, the equivalent series resistance (ESR) obtained from the X intercept of the curves [33] appears to be small at ~0.25 Ω for GO-OOH-N, which is slightly smaller than GO-N and GO-N-180, suggesting that GO-OOH-N electrode has lower resistance with good current response.

Figure 6f illustrates the Columbic efficiency of the GO-N and GO-OOH-N electrodes recorded during 500 continuous cycles at a current density of 1 A/g. After 300 cycles, the specific capacitance of GO-N and GO-OOH-N in 6 M KOH both retain approximately 90 % of the initial value. Moreover, the retention of specific capacitance of GO-N and GO-OOH-N decreases to 82.1 and 88.8 %, respectively. The promotion of the cycling performance of GO-OOH-N electrode reveals that a redistribution of oxygenic groups on precursor surface is an effective way to improve the stability of N-doped graphene during the charge/discharge process, due to a beneficial regulation on its chemical groups.

## Conclusions

In this article, the changes of N types in graphene were investigated by using different functionalized graphene oxide precursors under varied experimental conditions. The research result shows that low temperature was favorable for the generation of pyridone nitrogen, while high temperature was beneficial to the generation of pyridinic nitrogen. When GO-OOH was used as the precursor, the presence of carboxyl groups was favorable for the both generations of pyridinic nitrogen and pyridone nitrogen. The GO-OOH-N shows the highest capacitance, and an inferior of GO-OOH-N to GO-N in rate capability at high current density convinced the instability of pseudocapacitance in the fast charge/discharge process. Moreover, GO-OOH-N also showed an impressive specific capacitance and cyclic stability after 500 cycles at a discharge current density 1 A/g. These observations demonstrate that the regulation of nitrogen structures in graphene could be achieved by the well-controlled oxygen groups on graphene surface. We believe that the present way is beneficial for the supercapacitor applications or other fields.

## Additional file

Additional file 1: Figure S1. Dispersity of GO-N and GO-OOH-N solutions (in ethanol) placed for 3h. **Figure S2.** XPS C1s spectra of (a) G-OH; XPS N1s spectra of (b) G-OH-N and (c) GO-N-150. **Figure S3.** XPS O1s spectra of GO-N and GO-OOH-N. **Figure S4.** (a) Nitrogen adsorption/desorption isotherms and (b) pore size distributions of the GO-OOH-N and GO-N. **Table S1.** Elemental composition and distribution of type of carbon-containing groups on the surface of G-OH. **Table S2.** Elemental composition and distribution of the type of nitrogen-containing groups on the surface of G-OH-N samples. **Table S3.** Porous properties of GO-N and GO-OOH-N. **Table S4.** N-5 and N-6 content of N-doped graphene. (DOCX 2704 kb)

## Abbreviations

AFM: atomic force microscopy
CV: cyclic voltammetry
EDLC: electrochemical double-layer capacitors
EIS: electrochemical impedance spectroscopy
GO: graphene oxide
GO-N: N-doped graphene based on GO
GO-N-180: N-doped graphene based on GO at the react temperature of 180 °C
GO-OOH: carboxylic graphene
GO-OOH-N: N-doped graphene based on GO-OOH
PTFE: poly-tetrafluoroethylene
TEM: Transmission electron microscopy
XPS: X-ray photoelectron spectroscopy
XRD: X-ray diffraction
