# Supplementary material for: Carboxyl-Assisted Synthesis of Nitrogen-Doped Graphene Sheets for Supercapacitor Applications
Source: Nanoscale Res Lett. 2015 Aug 20;10:332. doi: 10.1186/s11671-015-1031-z (PMC4542773; doi:10.1186/s11671-015-1031-z)
Supplement: Additional file 1: Figure S1. — Dispersity of GO-N and GO-OOH-N solutions (in ethanol) placed for 3h. Figure S2. XPS C1s spectra of (a) G-OH; XPS N1s spectra of (b) G-OH-N and (c) GO-N-150. Figure S3. XPS O1s spectra of GO-N and GO-OOH-N. Figure S4. (a) Nitrogen adsorption/desorption isotherms and (b) pore size distributions of the GO-OOH-N and GO-N. Table S1. Elemental composition and distribution of type of carbon-containing groups on the surface of G-OH. Table S2. Elemental composition and distribution of the type of nitrogen-containing groups on the surface of G-OH-N samples. Table S3. Porous properties of GO-N and GO-OOH-N. Table S4. N-5 and N-6 content of N-doped graphene. (DOCX 2704 kb) [file 11671_2015_1031_MOESM1_ESM.docx]

**Additional file 1**

1. Synthesis of hydroxy graphene and N-doped graphene

**Hydroxy graphene (G-OH):** A 180 mg of graphene obtained from a thermal treatment on GO (1,000°C for 30 s) was dissolved into a 20.4ml methylbenzene. After an ultrasonic treatment for 45 min, the solution was washed with pentane and filtrated to obtain a black solid. Then, 105 mg of the black solid was dispersed into 35.1 ml of deionized water again by adding 52.5 mg of FeSO_4_ ·7H_2_O and 10.5 ml of H_2_O_2_ 30%). A well-dispersed solution was obtained after stirring for 12 h at room temperature. Centrifugal washing was then performed for several times with deionized water to remove the contaminants. Finally, the sample was dried under vacuum at 60°C.

**N-doped hydroxy graphene(G-OH-N):** 25mL of G-OH solution (6mg/ml) was taken and its pH was adjusted to 10 with NH_3_·H_2_O. Then 20mL of N_2_H_4_·H_2_O solution was added followed by stirring for 30 minutes. The solution was then transferred into the reaction vessel. After the reaction for 5h at 130 °C, black product was obtained. Centrifugal washing was performed for several times with distilled water and ethanol. G-OH-N was finally obtained by vacuum drying at 60°C.

**N-doped graphene based on GO (GO-N-150):** Process for preparation is the same as GO-N, except that the temperature of hydrothermal reaction is 150 °C.

2．Disperse ability of GO-N and GO-OOH-N


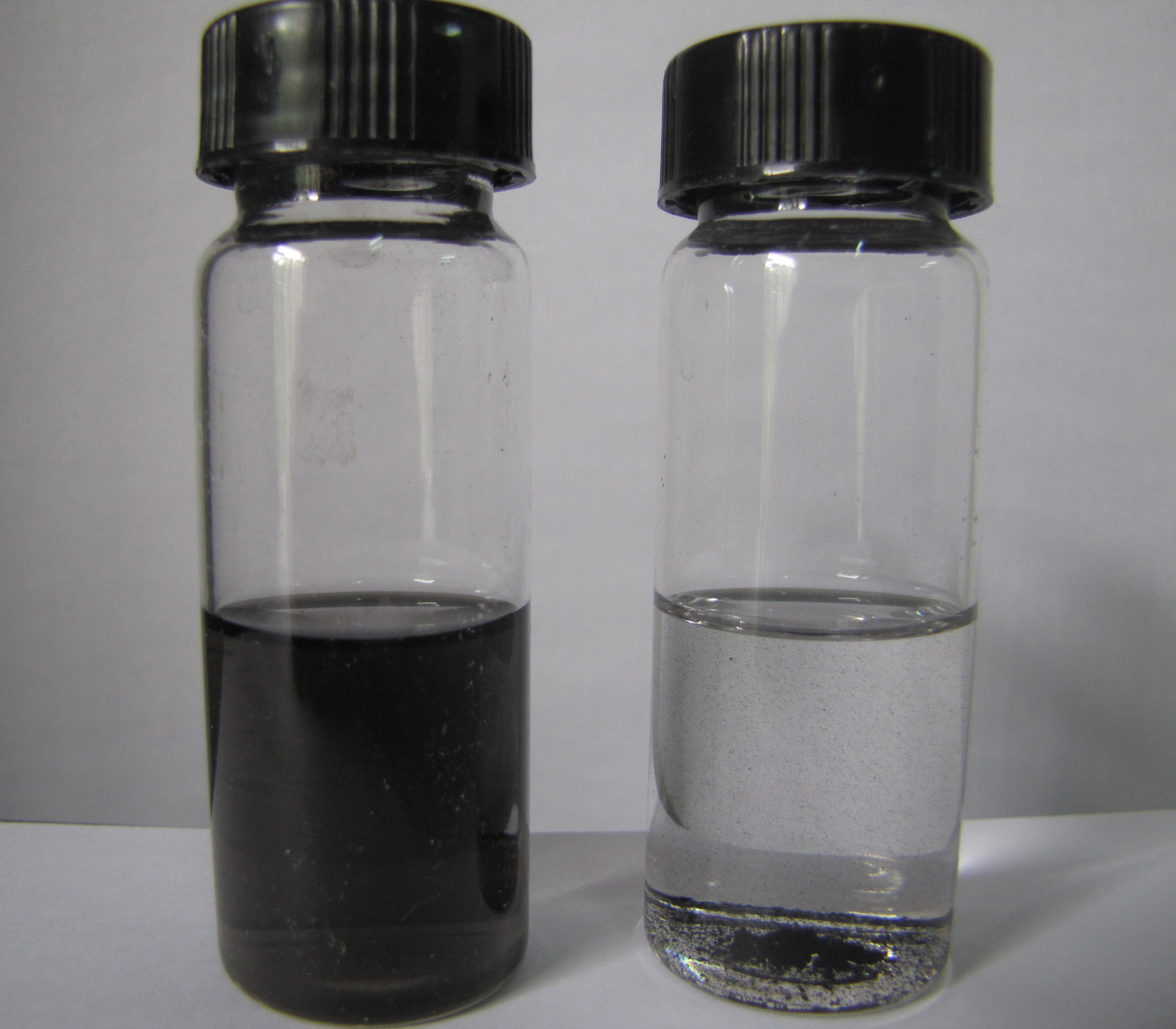


**GO-OOH-N**

**GO-N**

Fig S1 Dispersity of GO-N and GO-OOH-N solutions (in ethanol) placed for 3h.

3. XPS result of G-OH and G-OH-N


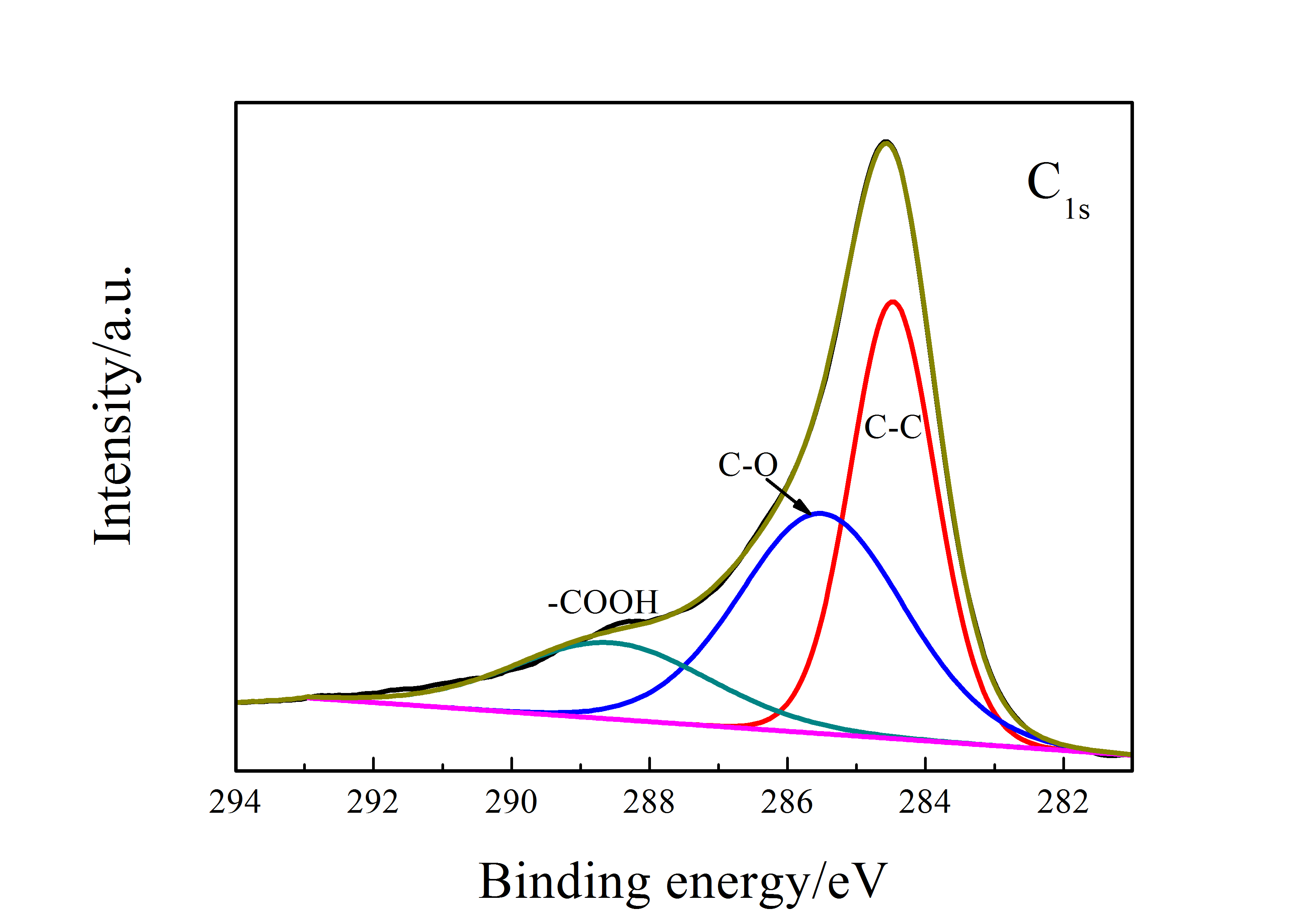


a


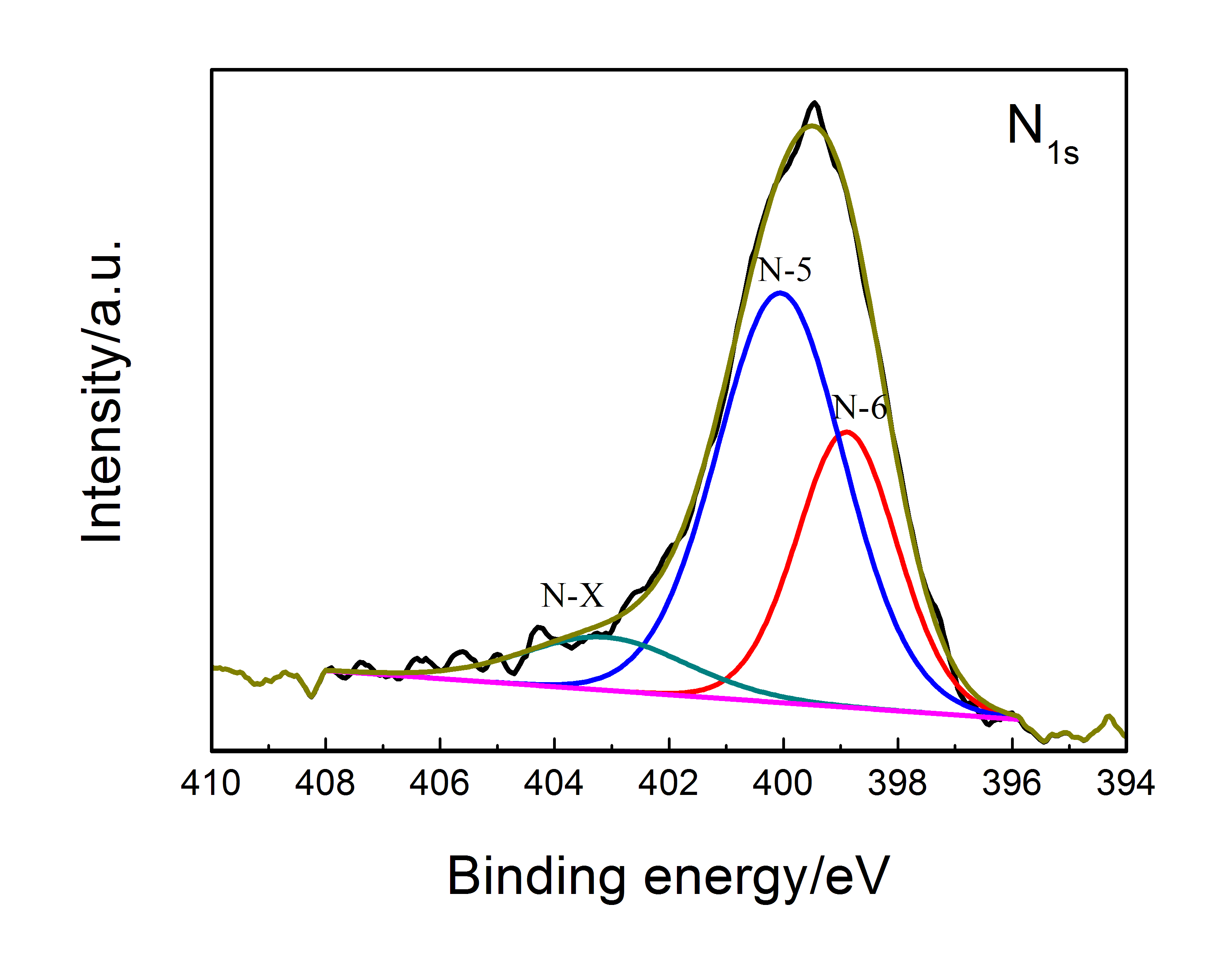


c


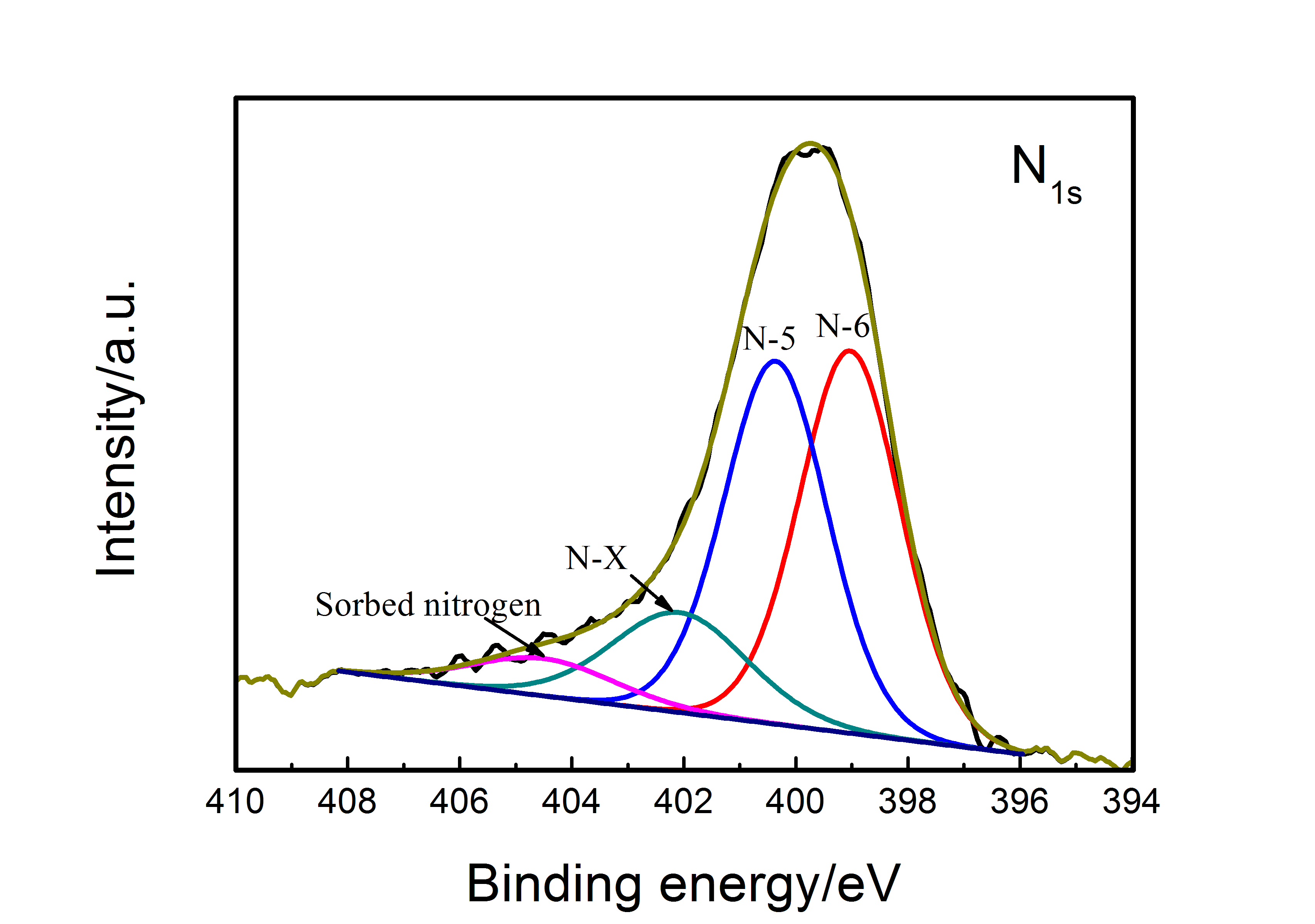


b

Fig S2 XPS C1s spectra of (a) G-OH; XPS N1s spectra of (b) G-OH-N and (c) GO-N-150


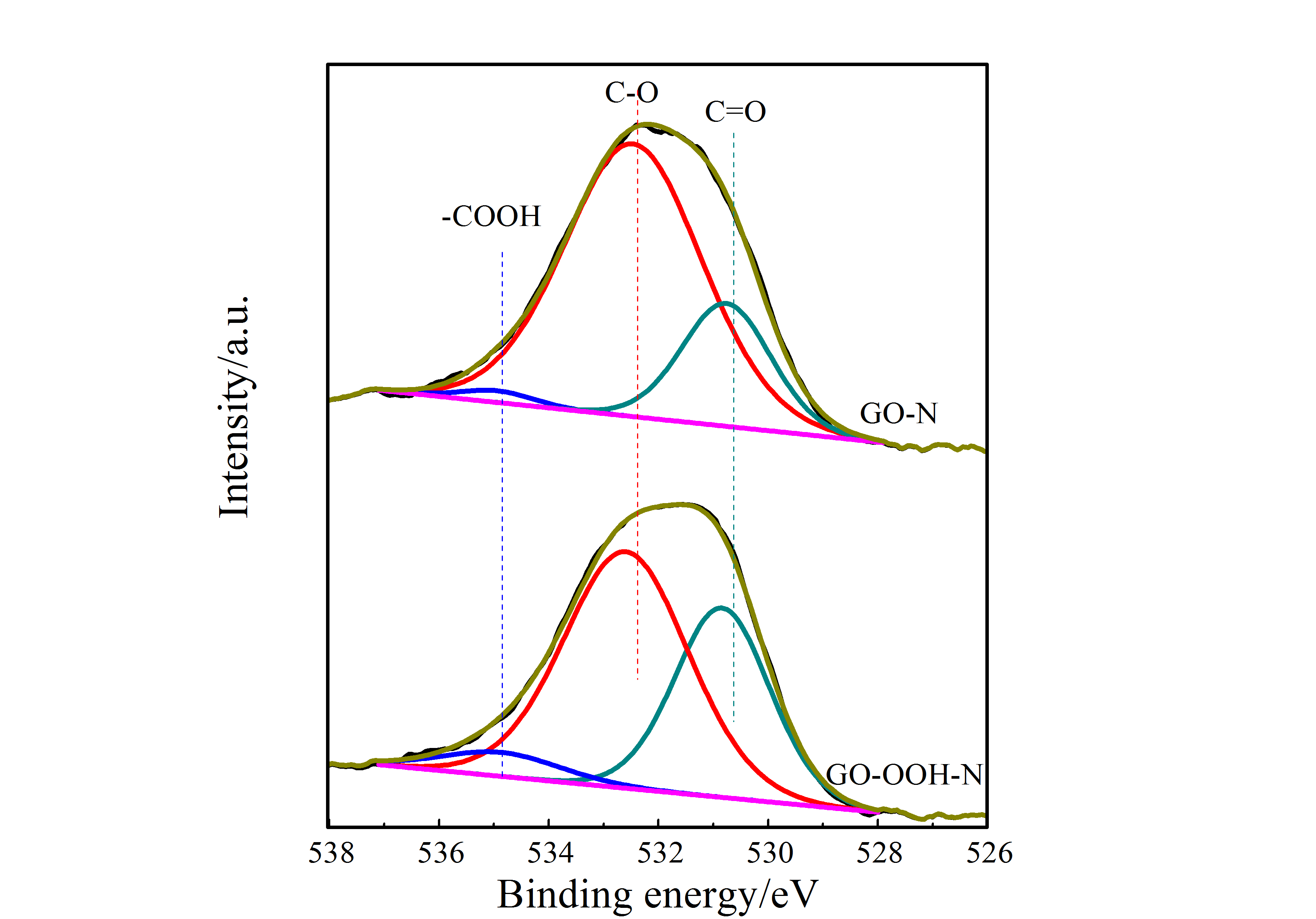


Fig S3 XPS O1s spectra of GO-N and GO-OOH-N.

(The main peak was divided into three sub-peaks according to ref.26)

**Table S1 Elemental composition and distribution of type of carbon-containing groups on the surface of G-OH**

| Sample | C/ at.% | O/ at.% | C/O | C distribution/ at.% | | | |  |
| --- | --- | --- | --- | --- | --- | --- | --- | --- |
|  | | | | C-C  (284.5eV) | C-O  (285.6eV) | -COOH  (288.6eV) |  | |
| G-OH | 70.8 | 29.2 | 2.4 | 29.8 | 29.0 | 12.0 |  | |

**Table S2 Elemental composition and distribution of the type of nitrogen-containing groups on the surface of**

**G-OH-N samples**

| Sample | C/ at.% | N/ at.% | N/C /% | N distribution/ at.% | | | | | | |  |
| --- | --- | --- | --- | --- | --- | --- | --- | --- | --- | --- | --- |
|  | | | | N-6  (399.0eV) | N-5  (400.1-400.4eV) | N-X  (402.1-403.1eV) | |  | |  |  |
| G-OH-N | 80.8 | 3.9 | 4.8 | 1.2 | 2.3 | | 0.4 | |  | | |
| GO-N-150 | 76.6 | 6.2 | 8.6 | 2.7 | 2.6 | | 0.9 | |  | | |

4. Porous properties of GO-N and GO-OOH-N


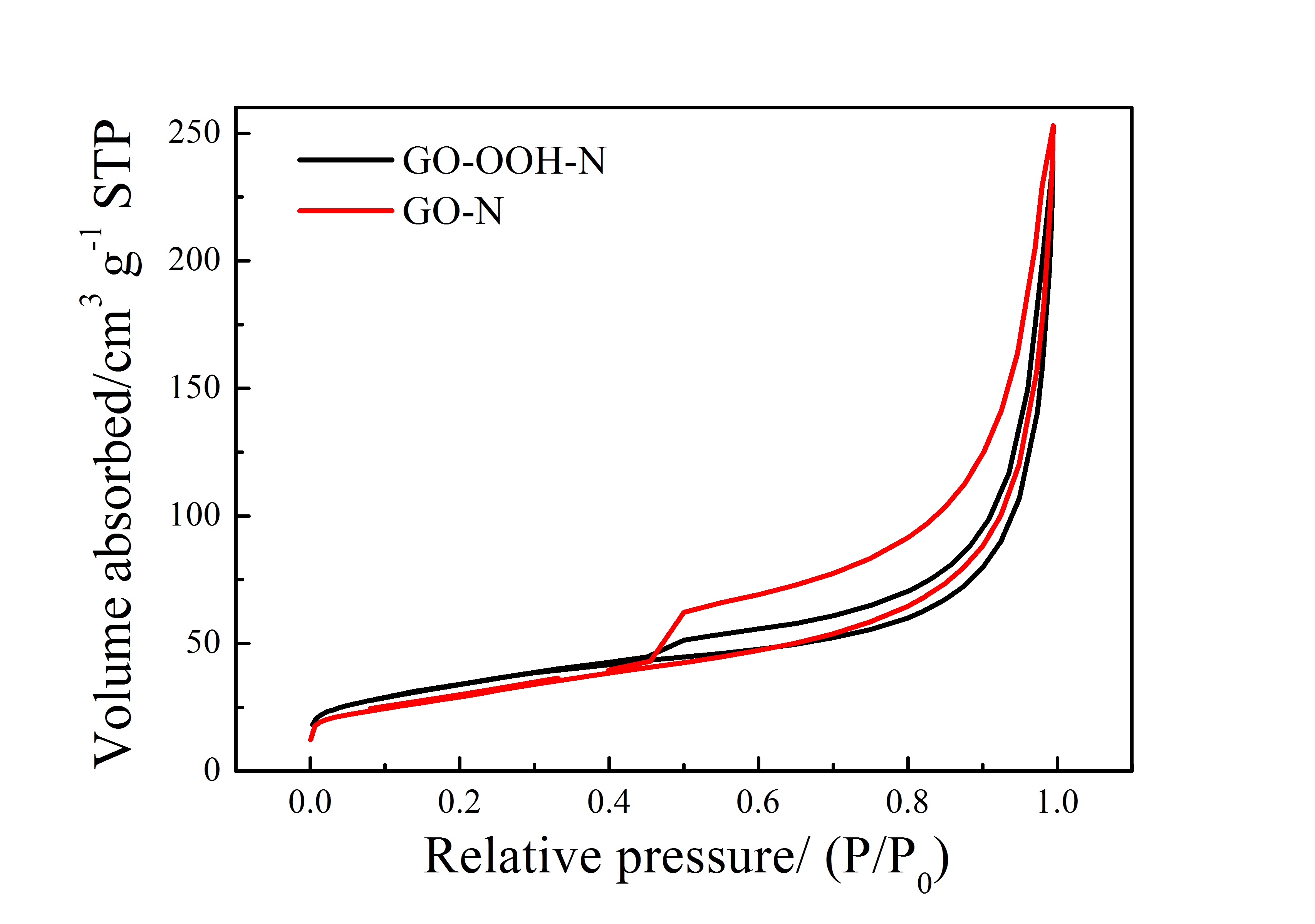

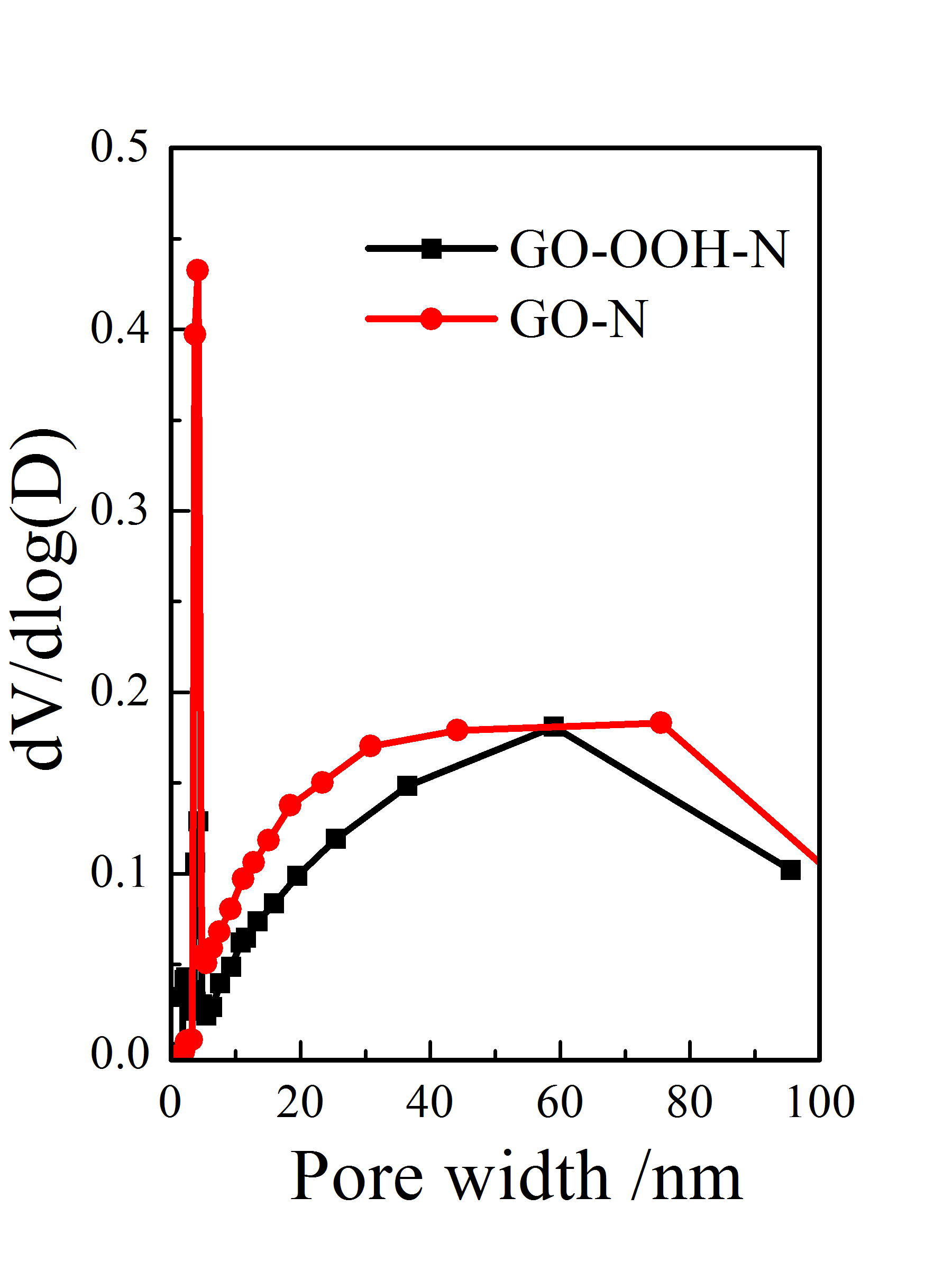


b

a

Fig.S4 (a) Nitrogen adsorption/desorption isotherms and (b) pore size distributions of the GO-OOH-N and GO-N.

**Table S3 Porous properties of GO-N and GO-OOH-N**

| Sample | S_BET_/m^2^/g | V_t_^a^/cm^3^/g | APD^b^/nm |
| --- | --- | --- | --- |
| GO-N | 262 | 0.225 | 14.2 |
| GO-OOH-N | 223 | 0.207 | 11.3 |

^a^ Single point total pore volume (V_t_) from adsorption isotherms at P/P_0_ ~ 0.99.

^b^Average pore diameter (4V/A by BET).

5. Comparison of N type and content of home-made N-doped graphene with others.

| Items | Methods/dopants | nitrogen contents(at. %) | N-5(%) | N-6(%) | N-5+N-6( %) | Ref. |
| --- | --- | --- | --- | --- | --- | --- |
| N-doped microporous  activated carbon | Stirred at room temperature/urea | 2.0 | 27.0 | 48.2 | 75.2 | 18 |
| NG | Hydrothermal(170℃.12h）/urea | 7.3 | 40.2 | 38.5 | 78.7 | 15 |
| NG | Hydrothermal(180℃.12h）/urea | 10.1 | 50.4 | 21.8 | 72.2 | 28 |
| NG | Microwave-assisted hydrothermal(190℃)/urea | 3.0 | 46.7 | 33.3 | 80.0 | 24 |
| NG | Microwave-assisted hydrothermal(180℃)/urea | 6.6 | 45.2 | 27.1 | 72.3 | 5 |
| NG | Microwave heating/NH_3_ | 5.5 | 30.1 | 43.6 | 73.7 | 36 |
| NG | Microwave-assisted hydrothermal/ammonia | 5.0 | 24.9 | 50.2 | 75.1 | 37 |
| NG | Hydrothermal(180℃.12h）/melamine | 25.3 | 12.2 | 57.3 | 69.5 | 38 |
| NG | Hydrothermal(80-200℃.3h）/N_2_H_4_ | 4.0 | 42.0 | 28.0 | 70.0 | 39 |
| NG | Annealing(550-1000℃)/NH_3_ | 5.2 | 53.1 | 18.5 | 71.6 | 40 |
| NG-OOH | Hydrothermal(130℃.5h）/N_2_H_4_ | 7.6 | 48.8 | 38.8 | 87.6 | paper |
| NG-OH | Hydrothermal(130℃.5h）/N_2_H_4_ | 3.9 | 59 | 30.8 | 89.8 | paper |

**Table S3 N-5 and N-6 content of N-doped graphene**

Reference

36. Wang Z, Li B, Xin Y, Liu J, Yao Y, Zou Z: **Rapid synthesis of nitrogen-doped graphene by microwave heating for oxygen reduction reactions in alkaline electrolyte**. *Chinese J Catal* 2014, 35:509-513.

37. Kim IT, Shin MW: **Synthesis of nitrogen-doped graphene via simple microwave-hydrothermal process**. *Mater Lett* 2013, 108:33-36.

38 Jiang Z-j, Jiang Z, Chen W: **The role of holes in improving the performance of nitrogen-doped holey graphene as an active electrode material for supercapacitor and oxygen reduction reaction**. *J Power Sources* 2014, 251:55-65.

39. Long D, Li W, Ling L, Miyawaki J, Mochida I, Yoon SH: **Preparation of nitrogen-doped graphene sheets by a combined chemical and hydrothermal reduction of graphene oxide**. *Langmuir* 2010, 26:16096-16102.

40. Lai L, Potts JR, Zhan D, Wang L, Poh CK, Tang C, Gong H, Shen Z, Lin J, Ruoff RS: **Exploration of the active center structure of nitrogen-doped graphene-based catalysts for oxygen reduction reaction**. *Energ Environ Sci* 2012, 5:7936.
